# Delayed Catastrophic Uterine Rupture in a Subsequent Pregnancy After Expectant Management of Placenta Accreta Spectrum

**DOI:** 10.7759/cureus.108494

**Published:** 2026-05-08

**Authors:** Nneoma B Edokobi, Tam D Nguyen, Kaitlin A Warta

**Affiliations:** 1 Department of Obstetrics and Gynecology, Novant Health New Hanover Regional Medical Center, Wilmington, USA; 2 Department of Minimally Invasive Gynecology Surgery, Duke University, Durham, USA

**Keywords:** abnormal placentation, high-risk obstetrics, maternal morbidity, obstetric complication, obstetric emergency, obstetrics and gynecology (ob/gyn), placenta accreta, placenta increta, placenta percreta, uterine conservation techniques

## Abstract

Placenta accreta spectrum (PAS) is a serious obstetric disorder involving abnormal placental attachment to the uterine wall and is associated with substantial maternal risk. While cesarean hysterectomy remains the standard treatment, conservative management approaches are emerging as alternatives. Minimal data is currently available regarding the long-term complications of conservative management. We present a case of a patient with a history of conservatively managed PAS who presented 14 years later during a subsequent pregnancy with fundal uterine rupture in the second trimester, highlighting the potential long-term risks associated with uterine sparing approaches.

A 39-year-old gravida 6 para 5003 woman presented at 26 weeks of gestation with acute abdominal pain and no fetal cardiac activity. Her history was notable for five prior cesarean deliveries, including a classical cesarean in her G4 pregnancy complicated by PAS. In that pregnancy, she underwent multidisciplinary conservative management, including classical cesarean delivery with the placenta left in situ and uterine artery embolization. Her postoperative course was uncomplicated, with successful uterine preservation. In her G6 pregnancy, due to concern for uterine rupture, decision was made to proceed with stat cesarean section. Upon entry into the abdominal cavity, she had experienced a fundal uterine rupture with complete placental separation. The site of the uterine rupture was able to be repaired, and the uterus was left in situ.

This case demonstrates that even when conservative, uterine-sparing management of PAS is pursued with careful patient selection and multidisciplinary planning, adverse outcomes can still occur. It highlights the need for long-term surveillance and further research to clarify the risks, outcomes, and reproductive implications of fertility-preserving approaches.

## Introduction

Placenta accreta spectrum (PAS) is a disorder that encompasses abnormal attachment and invasion of the placenta into the myometrium or surrounding extrauterine structures [1]. It includes placenta accreta, increta, and percreta, classified according to the depth of myometrial invasion, ranging from least severe (accreta) to most severe (percreta). PAS is typically diagnosed antenatally through imaging studies, with ultrasound (US) and magnetic resonance imaging (MRI), both of which have similar specificity and sensitivity for diagnosis of PAS [2,3]. The most significant risk factor for PAS is the combination of prior cesarean delivery and placenta previa [4]. More specifically, the likelihood of PAS increases with each additional cesarean delivery. A large multicenter study based in the United States of America demonstrated that among women with placenta previa, the risk of PAS rose incrementally from 3% with one prior cesarean, up to 11%, 40%, 61%, and 67% with two, three, four, and five or more previous cesarean deliveries, respectively [5]. Timely antenatal diagnosis of PAS is essential, as it significantly reduces the risk of postpartum hemorrhage, which is the most common maternal complication linked to PAS [6]. Maternal outcomes appear to improve when a cesarean delivery is followed promptly by a cesarean hysterectomy, provided the procedure is performed before the onset of labor [7]. While hysterectomy mitigates some risks, it does not eliminate them entirely. Significant concerns include the permanent loss of fertility, potential injury to pelvic organs, and the ongoing risk of severe hemorrhage. For some patients, these consequences may render the prospect of a total hysterectomy undesirable [8]. Newly emerging approaches to managing PAS involve leaving the placenta partially or entirely in situ, allowing conservation of the uterus. Conservative approaches to PAS can be grouped into four main strategies: extirpative management, expectant management, one-step surgery, and the triple P procedure. Extirpative management is when, after delivery, placental removal (manual or surgical) is attempted while preserving the uterus. This is often paired with aggressive hemorrhage-control measures (e.g., uterotonics, compression sutures, tamponade, vessel ligation/embolization) [9]. Expectant management, in contrast, leaves the placenta in situ after delivery (partial cord ligation with no traction), allowing the placental tissue to involute/resorb over time with close postpartum surveillance and readiness for delayed interventions (e.g., antibiotics, embolization, or delayed hysterectomy for hemorrhage/infection) [10]. One-step surgery involves en bloc resection of the invaded uterine segment with the placenta still attached and then immediate reconstruction of the uterus [11]. Lastly, “The Triple-P procedure” is an operation that includes perioperative localization of the placenta (to place the uterine incision above/away from the placenta), pelvic devascularization (often with prepositioned arterial occlusion balloons or other techniques), and placental non-separation with excision of the placental bed, followed by uterine repair [12]. All these strategies share the same goal: to minimize hemorrhage while preserving the uterus and future fertility. A 2025 systematic review and meta-analysis compared conservative approaches with cesarean hysterectomy across 16 comparative studies. Overall, conservative management was associated with significantly lower estimated blood loss and fewer genitourinary injuries. However, the review did not report an average duration of monitoring or follow-up for the conservative cohort, as outcomes were primarily perioperative and in-hospital. The authors emphasized the need for standardized reporting and longitudinal studies to better define long-term outcomes and refine patient selection [13].

## Case presentation

**Figure 1:**
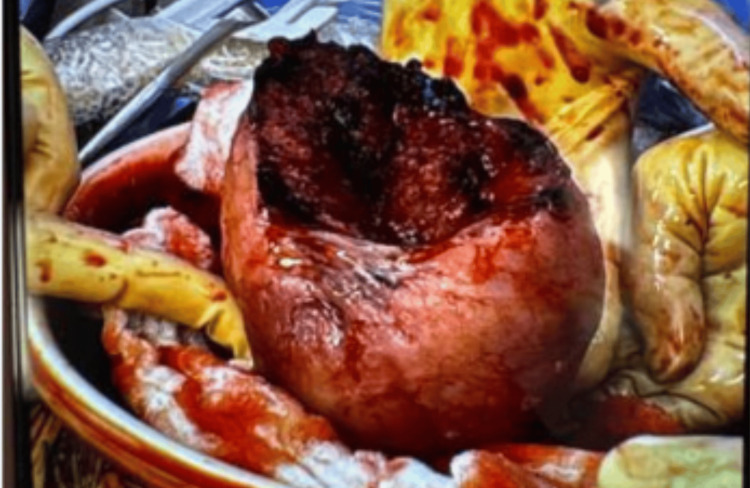
Intraoperative photo of the uterus with fundal rupture

A 39-year-old woman presented to labor and delivery triage via EMS at 26 weeks gestational age with a 48-hour history of abdominal pain that acutely worsened on the morning of presentation. Her medical history included substance use disorder (methamphetamine and tobacco) and anorexia, as well as five prior cesarean deliveries, including one classical incision. Upon arrival, no fetal heartbeat was detectable, although the patient reported feeling fetal movements earlier that day. Given the clinical suspicion of placental abruption versus uterine rupture, the patient underwent an emergent repeat cesarean section. Intraoperatively, approximately one liter of hemoperitoneum was noted, with a rupture of the uterine fundus (Figure 1); the placenta had completely separated from the uterus. The lower uterine segment was intact without evidence of a dehiscence. A nonviable infant was delivered. The uterine defect was repaired in three layers. Her postoperative course was uncomplicated. She was counseled on the risks of future pregnancy and declined both contraception and sterilization. She was subsequently lost to follow-up after discharge, with no postpartum visits completed.

Key factors increasing this patient’s clinical complexity included her extensive obstetric history. At her initial routine obstetric visit at 20 weeks’ gestation, she reported a history of only three prior low transverse cesarean deliveries, which was consistent with the records initially available at our institution's electronic medical record. She underwent an ultrasound, which demonstrated a normal-appearing and intact lower uterine segment without dehiscence. There was no sonographic evidence of placenta accreta. Estimated fetal weight was at the 85th percentile, and abdominal circumference was at the 96th percentile. Further research into the patient’s chart with Epic’s electronic medical records system, offering access to patient data from outside institutions, revealed a gravida 6, para 5003 obstetric history, including three prior low transverse cesarean sections performed at this institution and two cesarean sections at an outside facility, including one classical. The classical cesarean section was performed during her G4 pregnancy, 14 years prior, complicated by placenta previa with accreta and possible increta, as well as severe fetal central nervous system malformations. Then the patient presented to an outside facility where ultrasound revealed placenta previa complicated by placenta accreta and possible increta, as well as fetal holoprosencephaly, schizencephaly, agenesis of the corpus callosum, and absence of the septum pellucidum. She expressed a strong desire for fertility preservation and underwent extensive multidisciplinary counseling about the associated risks. She ultimately elected conservative uterine-sparing management, which involved preoperative placement of bilateral hypogastric artery balloons by interventional radiology, a planned classical cesarean delivery with the placenta left in situ, and subsequent embolization of uterine arteries to minimize placental blood flow. The patient experienced an uncomplicated postoperative course with minimal vaginal bleeding and no requirement for blood transfusion. She remained hospitalized for 10 days postoperatively for monitoring, during which time an ultrasound demonstrated appropriate placental involution. She was discharged home in stable condition with detailed emergency instructions and close outpatient follow-up arranged. Six years after her fourth pregnancy, she experienced an uncomplicated fifth pregnancy (G5), delivering a term infant via repeat cesarean section at the same outside hospital.

## Discussion

This case highlights the complex clinical decision making required in the management of PAS, particularly in patients with a strong desire for fertility preservation. Historically, the standard treatment for PAS has been cesarean hysterectomy, typically performed without attempting placental removal, which remains the definitive intervention for preventing massive hemorrhage and associated complications [14]. However, emerging data suggest that alternative approaches including conservative surgery and expectant management may be appropriate in select patients. These approaches aim to balance the risks of hemorrhage and organ injury against the desire to preserve future fertility. The patient in this case previously underwent a conservative uterine-sparing management strategy during her fourth pregnancy, which involved planned classical cesarean delivery with the placenta left in situ, prophylactic endovascular balloon placement, uterine artery embolization, and postoperative monitoring. Her successful outcome aligns with findings from recent literature indicating that, in carefully selected patients, conservative management can lead to favorable maternal outcomes with reduced intraoperative blood loss and avoidance of hysterectomy [11]. A large retrospective study from Indonesia comparing conventional hysterectomy with conservative surgical management in PAS found that conservative approaches were associated with shorter operative times, significantly less blood loss, and no maternal deaths in the conservative group, although patient selection likely favored lower-risk presentations [15]. Similarly, a pooled analysis of 12 studies involving 1,918 women demonstrated that expectant management was successful in preserving the uterus in approximately 68% of cases. However, up to 33% ultimately required hysterectomy within the first year postpartum, and serious complications such as infection, hemorrhage, and uterine necrosis occurred in about 6% of cases, most commonly in those requiring delayed hysterectomy [16]. The risk of failure and severe morbidity appears to correlate with the depth of placental invasion. Expectant management in placenta percreta, the most severe form of PAS, is associated with higher rates of delayed complications and treatment failure compared to placenta accreta or increta [17]. Despite promising outcomes in selected cases, conservative and expectant management strategies remain investigational and are not universally recommended. According to the American College of Obstetricians and Gynecologists and the Society for Maternal-Fetal Medicine, these approaches should only be considered in centers with appropriate multidisciplinary expertise and infrastructure to handle potential complications. Thorough counseling regarding the uncertain long-term outcomes, intensive follow-up requirements, and the potential for delayed life-threatening events is essential. Moreover, the long-term reproductive and gynecologic implications of conservative PAS management remain incompletely understood. Further research and longitudinal data are needed to clarify outcomes such as menstrual function, fertility, and the risk of complications in future pregnancies. As such, patients undergoing conservative treatment must be closely monitored postpartum with imaging and clinical follow-up to promptly identify and manage delayed complications.

## Conclusions

While conservative management of PAS, including leaving the placenta in situ, offers a fertility-sparing alternative to hysterectomy, it requires meticulous patient selection, informed consent, and comprehensive long-term surveillance. In this case, the patient’s strong desire for future fertility, multidisciplinary planning, and initially favorable course highlight how conservative PAS management can be successful in a highly controlled setting. However, once patients transition out of close follow-up, long-term surveillance and adherence are less certain, increasing the risk of delayed adverse outcomes. More data are needed to better define the long-term ramifications of conservative management strategies for PAS.
